# Mutation in the *xpsD* gene of *Xanthomonas axonopodis* pv. *citri* affects cellulose degradation and virulence

**DOI:** 10.1590/S1415-47572009005000110

**Published:** 2010-03-01

**Authors:** Juliana Cristina Baptista, Marcos Antonio Machado, Rafael Augusto Homem, Pablo Sebastián Torres, Adrian Alberto Vojnov, Alexandre Morais do Amaral

**Affiliations:** 1 Centro APTA Citros Sylvio Moreira, Cordeirópolis, SPBrazil; 2 Universidade Estadual de Campinas, Campinas, SPBrazil; 3 Fundación Pablo Cassará, Instituto de Ciencia y Tecnología Dr. Cesar Milstein, Ciudad de Buenos AiresArgentina; 4 EMBRAPA Recursos Genéticos e Biotecnologia, Brasília, DFBrazil

**Keywords:** citrus canker, transposome, type II secretion system

## Abstract

The Gram-negative bacterium *Xanthomonas axonopodis* pv. *citri*, the causal agent of citrus canker, is a major threat to the citrus industry worldwide. Although this is a leaf spot pathogen, it bears genes highly related to degradation of plant cell walls, which are typically found in plant pathogens that cause symptoms of tissue maceration. Little is known on *Xac* capacity to cause disease and hydrolyze cellulose. We investigated the contribution of various open reading frames on degradation of a cellulose compound by means of a global mutational assay to selectively screen for a defect in carboxymethyl cellulase (CMCase) secretion in *X. axonopodis* pv. *citri*. Screening on CMC agar revealed one mutant clone defective in extracellular glycanase activity, out of nearly 3,000 clones. The insertion was located in the *xpsD* gene, a component of the type II secretion system (T2SS) showing an influence in the ability of *Xac* to colonize tissues and hydrolyze cellulose. In summary, these data show for the first time, that *X. axonopodis* pv. *citri* is capable of hydrolyzing cellulose in a T2SS-dependent process. Furthermore, it was demonstrated that the ability to degrade cellulose contributes to the infection process as a whole.

## Introduction

Citrus canker is a highly destructive disease of citrus crops, which occurs worldwide and affects most of the commercial varieties of citrus. The disease is caused by the bacterium *Xanthomonas axonopodis* pv. *citri*. In areas where citrus canker has been established, its control is very difficult. Control is extremely difficult in areas where the disease is already firmly established, and is based on eradication programs involving the heavy use of copper compounds. Bacterial plant pathogens, such as xanthomonads deliver various virulence proteins that play a role during infection. Knowledge on the complete repertoires of specific types of effectors would be beneficial towards understanding the mechanisms by which bacteria interact with plants, thereby causing diseases (Collmer *et al.*, 2002, Guttman *et al.*, 2002, Roden *et al.*, 2004, Chang *et al.*, 2005, Nomura and He, 2005).

The complete genome sequencing of *X. axonopodis* pv. *citri* revealed several open reading frames that were annotated as related to pathogenicity and virulence (da Silva *et al.*, 2002). Unexpectedly, given that the symptoms caused by *X. axonopodis* pv. *citri*, a leaf-spot pathogen, are not typically associated to massive degradation of the plant-cell wall, such sequences include a number of genes that share similarity with those that code for plant cell wall degradation in other organisms. Plant cell walls are comprised mainly of cellulose and hemicellulose as major components, and also lignin, pectin, among others. Apparently there is no correlation between citrus canker symptoms and citrus tissue degradation caused by cell-wall cleavage. Interestingly, *X. axonopodis* pv. *citri* was found to express cell-wall degrading enzymes when grown in a medium that supposedly mimics the environment of plant intercellular spaces (Astua-Monge *et al.*, 2005).

In *Erwinia* species, plant-cell wall cleavage is a key step for the pathogenesis. In fact, virulence depends basically on the production and secretion of host-cell wall degrading enzymes (Matsumoto *et al.*, 2003, Corbett *et al.*, 2005). To date, most of the proteins that have been characterized in bacterial plant pathogens, and also involved in the degradation of several components of plant-cell walls, are secreted by the type II secretion system (T2SS) (Jha *et al.*, 2005). A functional T2SS is known to be required for virulence and the secretion of cell-wall degrading enzymes, in at least two xanthomonads that are vascular pathogens in their plant hosts, *Xanthomonas oryzae* pv. *oryzae* and *Xanthomonas campestris* pv. *campestris* (Chen *et al.*, 2005; Lee *et al.*, 2005; Jha *et al.*, 2007).

Although *X. axonopodis* pv. *citri* harbors the T2SS, little is known regarding the effects of this system and the proteins that are delivered during its action. This study reports on the role of *xps*D, a T2SS component, in the ability of *X. axonopodis* pv. *citri* to cause the disease and degrade cellulose.

## Materials and Methods

###  Bacterial strains, plasmids and growth conditions

The bacterial strains and plasmids used in the present study are listed in Table 1. *Xanthomonas axonopodis* pv. *citri* 306 strain, already described (da Silva *et al.*, 2002), was routinely cultured on nutrient broth (NB) medium [containing per liter, 15 g of peptone, 3 g of yeast extract, 6 g of NaCl and 1 g D(+) glucose] at 28 °C, while *Escherichia coli* cultures were grown on Luria-Bertani medium at 37 °C. Kanamycin (15 μg/mL) was used for selection in *E. coli* and *X. axonopodis* pv. *citri*. The plasmid for site-directed mutagenesis was prepared from the *E. coli* host strain by the alkaline lysis method.

###  DNA manipulation

Insertion points were confirmed by DNA sequencing with the forward and reverse primers of EZ::TN (Epicentre Technologies, Madison, WI) (Table 2). For genomic DNA isolation, strains were cultured overnight in 50 mL of NB. Cultures were then adjusted to optical density of 0.6 at 600 nm in sterile NB, and DNA was isolated using the CTAB (hexadecyltrimethylammonium bromide) method (Ausubel *et al.*, 1998). DNA was stored in Tris-EDTA buffer (10 mM Tris, 1 mM EDTA, pH 8.0) at -20 °C. For Southern blotting and hybridization, 2 μg of genomic DNA from *X. axonopodis* pv. *citri* were digested with *Eco*RI and *Eco*RV, separated by electrophoresis in an 0.8% agarose gel, and transferred onto Hybond-N nylon membrane (Amersham Pharmacia Biotech, Little Chalfont, Buckinghamshire, England) in 5 M NaOH, with subsequent cross-linking by exposure to UV irradiation. Hybridization was carried out at 65 °C. Southern blotting analysis was performed with digoxigenin (DIG)-labeled PCR products corresponding to a 399-bp fragment of *aph* gene as probe, by using the PCR DIG Probe Synthesis Kit and DIG DNA Labeling and Detection Kit (Roche Diagnosis, Indianapolis, IN). The analysis was used to confirm single EZ:TN insertions and homologous recombination.

###  Construction of mutant strains

For random insertion mutagenesis, the EZ::TN < KAN-2 > transposome complex, a mixture of the transposon EZ::TN < KAN-2 > and EZ::TN transposase (Epicentre Technologies, Madison, WI), was introduced by electroporation directly into *X. axonopodis pv. citri* 306 electrocompetent cells. These cells were prepared as described by Amaral *et al.* (2005). A Gene Pulser II Electroporator (Bio-Rad, Hercules, CA) was used to transform 50 μL of cell suspension with 1 μL of the transposome complex, under the following conditions: 50 Ω, 50 μF, and 2.5 kV in a 0.2-cm cuvette. After electroporation, cells were suspended in 1 mL of SOC broth (containing, per liter, 20 g of tryptone, 5 g of yeast extract, 0.5544 g of NaCl, 0.1864 g of KCl, 1.2038 g of MgSO_4_, 0.9522 g of MgCl_2_ and 3.2 g of glucose) and allowed to recover for 2-3 h at 28 °C with shaking. To confirm the phenotypes, the site-directed Δ*xpsD* mutant was produced by using the primers XpsD_EcoRI_F and XpsD_EcoRI_R to PCR amplify an internal fragment of the *xps*D region from *X. axonopodis* pv. *citri* 306 genomic DNA. The 583-kb PCR product was cloned into the pCR2.1-TOPO vector (Invitrogen, Carlsbad, CA), thereby generating TOPO-*xps*D, and then electroporated into *E. coli*, whereupon the plasmid DNA was extracted and electroporated into *X. axonopodis* pv. *citri*.

###  Plate assay

Carboxymethyl cellulose (CMC) (0.5%) was incorporated into agar plates with culture medium (0.1% NaNO_3_, 0.1% K_2_HPO_4_, 0.1% KCl, 0.05% MgSO_4_, 0.05% yeast extract, 0.1% glucose and 1.7% agar). Colonies were grown at 28 °C for 48 h and then washed off with water. The plates were flooded with 1% Congo Red for 30 min and washed with 1 M NaCl. CMC degradation was detected by yellow halos under and around the colonies, and CMCase activity analyzed by using the (H^2^ - C^2^)/ C^2^ ratio (H - diameter of the halo, C - diameter of the colony). For each strain, the hypothesis of equivalent CMC degradation in the two groups (wild-type and mutant strains) was tested by means of the Student two-sample *t* test.

###  DNA sequencing

The general PCR procedure has been described by Sambrook *et al.* (1989). For mapping the location of transposon insertion, TAIL-PCR (thermal asymmetric interlaced PCR) was performed to amplify unknown DNA sequences contiguous to known *kan* gene sequences, according to the method of Liu and Whittier (1995). Three successive high- and low-stringency PCR amplifications were performed with nested sequence-specific primers and shorter arbitrary degenerate primers with genomic templates from the mutant strains generated by random insertion. Sequences were compared and aligned with sequences from the GenBank database, by using the BLAST program of the National Center for Biotechnology Information website.

###  Sodium dodecyl sulfate-polyacrylamide gel electrophoresis (SDS-PAGE)

For comparative analysis of proteins secreted by both *X. axonopodis pv. citri* 306 and Δ*xpsD* mutant strains, the bacteria were grown for 24 h at 28 °C in XMV2 medium (Schulte and Bonas, 1992) to an optical density of 0.6 and the culture-supernatant proteins were concentrated by precipitation with 10% trichloroacetic acid, as previously described (Economou *et al.*, 1990), except that after precipitation, the trichloroacetic acid was extracted by washing the precipitate with acetone. Proteins from an equivalent of 10 mL of culture supernatant were separated by SDS-PAGE (Bradley *et al.*, 1988) with 12% acrylamide and visualized by staining with Silver Stain Kit (Bio-Rad, Hercules, CA).

###  Plant material and plant inoculation

Pathogenicity and virulence assays were performed by using sweet orange cv. Baia (*Citrus sinensis* L. Osbeck) as host of all mutant strains. All plants were grown in growth chambers at 28 °C with a 12-h photoperiod. Inoculum concentrations were adjusted to an optical density of 0.6 at 600 nm. For pathogenicity and virulence tests, plants were inoculated by syringe infiltration with needle and, to mimic the natural infection process (bacteria entering the plant through the stomata), leaves were sprayed with the bacterial suspension on the abaxial surface. Symptoms were scored by any visual modification on lesion. Bacterial growth in citrus leaves was measured by harvesting leaf discs for each *X. axonopodis* pv. *citri* mutant strain. The leaves were ground with a mortar and pestle in 1 mL of 0.01 M phosphate buffer, pH 7.2. The solution was serially diluted and spread onto NB plates, with proper antibiotics. The mean number of colonies in plates of the proper dilutions was calculated.

## Results

###  Construction of a knockout mutant library and isolation of cellulose degradation deficient *X. axonopodis* pv. *citri* mutants

To confirm the presence of functional genes for CMC degradation in *X. axonopodis* pv. *citri*, a collection of nearly 3,000 knock-out mutants was produced by random insertion of a cassette for resistance to kanamycin.

**Figure 1:**
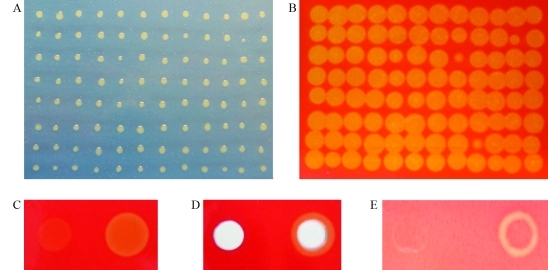
Selection of *X. axonopodis* pv. *citri* mutant colonies defective in CMC degradation. In panel A, Petri dish with 96 colonies grown on CMC medium, 2 days after inoculation; B, Halo of CMC degradation in Petri dish after colonies were washed off and Congo red staining was applied. C. Agar medium topped with CMC was seeded with lawn of mutant strain 30E9 (left) and wild-type (right) (40 μL per spot), grown for 24 h, washed off and then stained with Congo red. In panel D, the same procedure as that in panel C, however wells were made in the medium before seeding the cells. In panel E, foliar discs of citrus plants were infiltrated, placed onto the medium and then removed after 24 h, whereupon plates were stained. All strains were grown at 28 °C in NB liquid medium and harvested at O.D._600_ = 0.5 for inoculation. Unstained regions correspond to areas where CMC has been degraded.

To evaluate the capacity of *X. axonopodis* pv. *citri* in hydrolyzing cellulose-containing material, we performed a global screening to search strains for lack-of-function (Figure 1 A and B). Individual transformants from the random mutant libraries were replicated onto squared CMC plates for phenotypic screening.

Carboxymethyl cellulose (CMC) is a high-molecular weight polymer that mimics cellulose without being transported into cells (Kim *et al.*, 2000). Cell colonies that hydrolyze CMC can be clearly identified by the surrounding halo after treatment with Congo red. For more reliable screening of mutants with modified CMCase activity, selection criteria were changed from the intensity of the clear zones apparent below transformant colonies on CMC plates, to the size of clear-zone formation around the colonies, thereby obtaininga few candidate mutant strains.

From the knock-out mutants produced by random insertion, the transformant named 30E9 exhibited a smaller, superficial clear zone on the CMC plate, suggesting that it is merely partially functional (Figure 1C and D). The ability of the mutant 30E9 to promote CMC degradation from agar plates was very weak and limited, only occurring below the colony. This inconsiderable CMC degradation remained stable for several days after inoculation. Areas with CMC degradation were significantly lower on spots with the 30E9 mutant strain than on those with the wild-type (*t* test with paired two samples for mean, p < 0.001; *n* = 24 spots analyzed per each strain) (Table 3). To check whether the CMC degradation in the mutant strain could be induced by the plant tissue, infected foliar discs were placed on a CMC plate (Figure 1D). Despite the fact that the wild-type strain showed CMCase activity below the edge of the discs, the mutant strain had little effect.

**Figure 2:**
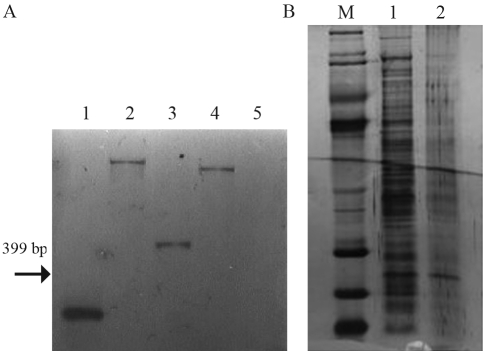
A. *Southern blot* by using a fragment of *npt*II (399 pb) as a probe (1) to confirm single insertion. Total genomic DNA of the strain 30E9 (*xps*D) from *Xac* 306 digested with restriction enzymes (2: *Eco*RI, 3: *Eco*RV, and 4: *Nc*OI) and wild strain (5). B. SDS-PAGE of the secreted proteins (supernatant) from *Xanthomonas axonopodis* pv. *citri* in the XVM2 medium. Lane 1, molecular mass marker; lane 2, wild-type strain; lane 3, xpsD defective strain.

To identify the site of transposon integration, we determined the DNA sequence adjacent to the *kanR* cassette for this strain. The 30E9 mutant had inactivation of the gene *xps*D (GenBank accession n. AAM38377.1), with the transposon located at the position 4,178,942 in the genome (GCACGATG < KAN-2 > TCCAGAA). Since the transposon EZ::TN < KAN-2 > shows no origin of replication it cannot be rescued by digesting genomic DNA of the mutant strain, therefore the TAIL-PCR technique was used to map the site of cassette insertion. Southern blot analysis verified that a single insertion was responsible for the phenotype (Figure 2A).

###  Interruption of *X. axonopodis* pv. *citri xps*D gene results in the modification of bacterial secretion, disease symptoms and multiplication on citrus leaves

**Figure 3:**
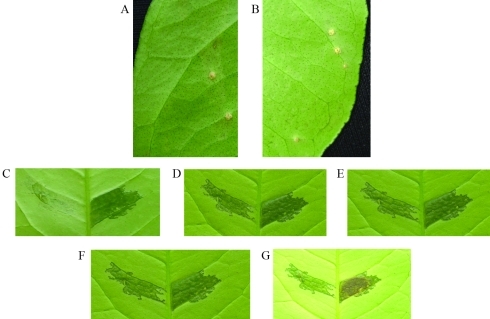
Foliar symptoms of inoculation with *X. axonopodis* pv. *citri* in citrus. In panels A (mutant strain) and B (wild-type strain), symptoms of spray inoculation with Xac (O.D._600_ = 0.5) on the abaxial surface of sweet orange leaves. The photographs were taken 30 days post-inoculation. In panels C, D, E, F and G, the strains were inoculated (O.D._600_ = 0.5) into the side of the mid-vein (mutant strain, left; wild-type strain, right), and respective photographs taken 2, 4, 6, 8 and 10 days post-infiltration, in each case.

**Figure 4:**
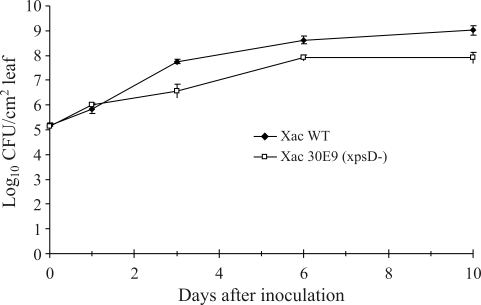
Growth curve of *Xanthomonas axonopodis* pv. *citri* (Xac) strains on leaves of susceptible sweet orange cultivar ‘Baia' 0, 1, 3, 6 and 10 days after inoculation by syringe infiltration with needle. Xac WT: wild-type strain; Xac 30E9 (xpsD-): *xps*D mutant strain. Data represent the means of three assays.

The screening of the transposon library led to the identification of a clone with an impaired ability to hydrolyze cellulose. Also, the lack-of-function for the gene investigated in this study had effect on bacterial secretion (Figure 2B), disease symptoms (Figure 3 A-E), and growth *in planta* (Figure 4). Nevertheless, no apparent differences in symptom severity were observed on comparing mutant and wild-type strains when the inoculation method (spray) reproduced natural-infection phenomena (Figure 3F and G).

As shown by protein profiles (Figure 2B), CMCases certainly are not the only enzymes whose secretion is affected by mutation in *xps*D of *X. axonopodis* pv. *citri*. In fact, the *xps*D gene characterized in *X. campestris* pv. *campestris* was involved in the secretion of a number of extra-cellular enzymes (Hu *et al.*, 1992).

To confirm that during infection on leaves, the transposon was not excised from the bacterium due to selection, the strains were re-isolated from the infected tissues and plated using an appropriate antibiotic. Unlike the wild-type strain, the mutant strain was capable of growing on plates with kanamycin, and the 399-bp fragment of *aph* (kanamycin-resistance gene) was amplified by PCR.

A clear difference in symptomatology was found according to the method of inoculation used in the study. Although no differences were detected in leaves inoculated by spray (Figure3A and B), we found clear altered symptoms in the inoculated zone of the mutant strain compared to the wild-type strain in infiltrated leaves (Figure 3C to G). The differences were especially notable at the first two days after inoculation (Figure 3C), by a delay of appearance of symptoms in the mutant strain, and at ten days (Figure 3G), when the wild-type strain showed necrosis in the inoculated zone, in opposition to the mutant strain.

To ascertain the influence of the *xps*D genes in citrus canker development, the growth of *X. axonopodis* pv. *citri* was compared and differences between the two strains observed (Figure 4). A smaller population of the mutant strain was observed within 3 days after inoculation and remained until the stationary phase.

## Discussion

In this study, we demonstrate that the T2SS exerts an influence on the ability of *X. axonopodis* pv. *citri* to colonize host tissues and is able to mediate the hydrolysis of cellulose. *X. axonopodis* pv. *citri* enters citrus tissues through stomata and wounds, and has been traditionally described as causing lesions where the center becomes raised and spongy, or corky (Graham *et al.*, 1992; Graham *et al.*, 2004), aspects not typically related to symptoms of plant-cell degradation. However, the results presented above showed that *X. axonopodis* pv. *citri* cells have the ability to hydrolyze cellulose.

The lack of a halo of CMC degradation around colonies of *X. axonopodis* pv. *citri* was expected to result from the inactivation of either a gene for a major cellulose-degrading enzyme (Walker *et al.*, 1994), a gene-regulator (Vincent-Sealy *et al.*, 1999), or a component of the secretion system itself (Ray *et al.*, 2000). The fact that the scant CMC degradation on agar plates only occurred below the colony, and furthermore that no surrounding degradation halo was detected through staining, all implies that the mechanism is cell bound (Zorreguieta *et al.*, 2000).

Studies indicated that when the *xps*D gene was interrupted, extracellular enzymes polygalacturonate lyase, alpha-amylase and endoglucanase accumulated in the periplasm of *Xanthomonas campestris* pv. *campestris* (Hu *et al.*, 1992). Likewise, our study has demonstrated by protein profile that *X. axonopodis* pv. *citri* which lacks a functional *xps*D gene was clearly affected in its secretion.

XpsD is the outer membrane single protein of the xps cluster-encoded T2SS in Gram-negative bacteria, which requires a multicomponent assembly apparatus for the secretion of extracellular enzymes. Recent articles on several plant pathogens report a key role of the xps cluster during pathogenesis (Jha *et al.*, 2005, 2007). This finding has shed light on the possibility of *X. axonopodis* pv. *citri* being capable of somehow modifying cellulose components in its environment, and is consistent with the effects of the T2SS found in other bacteria (Ray *et al.*, 2000; Zhou and Ingram, 2000; Zorreguieta *et al.*, 2000; Jha *et al.*, 2005, 2007). In fact, *X. axonopodis* pv. *citri*, like *X. campestris* pv. *vesicatoria* and *X. campestris* pv. *campestris*, harbors two gene clusters coding for different T2SSs (the xcs and xps type II secretion systems), whereas *X. oryzae* pv. *oryzae* encodes only the xps cluster. So far, there are no reports on the role played by the xcs-encoded T2SS in xanthomonads.

We had no means of complementing *X. axonopodis* pv. *citri* to evaluate whether wild-type *xpsD* genes are capable of inducing virulence and cellulose degradation, since amplification of the full gene (2,291 bp in length) from the genome or cosmid has so far been unsuccessful. Thus, complementation tests were not accomplished.

Although it remains unknown whether the degradation of cellulose can be unequivocally imputed to a major gene or is the result of the joint-action of a number of genes, it seems clear that such activity is mediated by the T2SS. Likewise, the same machinery is in some way responsible for the secretion of proteins that play important roles in virulence.

In *Erwinia chrysanthemi*, two endoglucanases (CelZ and CelY) are produced, however CelZ represents approximately 95% of the total carboxymethyl cellulase activity (Zhou and Ingram, 2000). The particular *X. axonopodis* pv. *citri* enzymes involved in cellulose hydrolysis have not, as yet, been identified. Even though many more insertion strains must be tested for this screen to reach saturation, these results suggest that the proteins related to CMC degradation seem to be exclusively secreted by the *xps*-encoded T2SS present in *X. axonopodis* pv. *citri*. Furthermore, even though incapable strains represent only a small fraction of the total number of sequences annotated as putatively involved in the process, this mutant strain could possibly make a significant difference for studies involving plant cellulose and the action of plant pathogens.

The multiplication of *X. axonopodis* pv. *citri* was significantly suppressed when inoculated into sweet orange leaves. The observation of reduced disease severity following inoculation with the 30E9 was different from that observed when *xps*D-defective *X. campestris* pv. *campestris* was inoculated into cabbage (Hu *et al.*, 1992). In fact, apart from these two pathogens infecting distinct plant hosts, a major difference between these xanthomonads is their mode of action. Whereas *X. axonopodis* pv. *citri* is a leaf-spot pathogen, *X. campestris* pv. *campestris* is a vascular bacterium. Moreover, in the spray process emulating natural infection, there were no visible modifications in lesions. Collectively, these data may indicate specific roles played by the T2SS according to the pathosystem.

The work described herein was designed to test the ability of *X. axonopodis* pv. *citri* in using the T2SS during infection and cellulose hydrolysis, in view to the adequate application of potential disease-control strategies. In summary, we have identified that *X. axonopodis* pv. *citri* is capable in hydrolyzing cellulose and that its T2SS has effects on the disease symptoms. These data demonstrate that the T2SS present in *X. axonopodis* pv. *citri* plays various and substantial biological and ecological roles that remain to be elucidated.

## Figures and Tables

**Table 1:** Bacterial strains and plasmids.

Strain or plasmid	Characteristics	Source or reference
*Escherichia coli*		
DH5α	F^-^ φ80d*lacZ*ΔM15 Δ (*lacZYA-argF*)*U169 endA1 deoR recA1 hsdR17*(r_K_^-^ m_K_^+^) *phoA supE44* λ^-^*thi-1 gyrA96 relA1*	Gibco

*Xanthomonas axonopodis* pv. *citri*		
306	Wild type, Ap^r^	R.P. Leite
30E9	Kan^r^, XpsD-	This study
Δ*xpsD*	Kan^r^, site-directed XpsD-	This study

Plasmid		
pCR2.1-TOPO	vector pUC18 derivative, kanr, *bla* (Apr), *lac*Z	Invitrogen
TOPO-*xpsD*	PCR-amplified *xpsD* (725-1307) in pCR2.1-TOPO; Kan^r^	This study

**Table 2:** Oligonucleotides used.

Primer	Nucleotide sequence (5' → 3')^a^
KAN RP-1	GCAATGTAACATCAGAGATTTTGAG
KAN2 FP-1	ACCTACAACAAAGCTCTCATCAACC
KanA	CATGCAAGCTTCAGGGTTGA
AD1	NTCGA(G/C)T(A/T)T(G/C)G(A/T)GTT
AD2	NGTCGA(G/C)(A/T)GANA(A/T)GAA
AD3	(A/T)GTGNAG(A/T)ANCANAGA
AD4	AG(A/T)GNAG(A/T)ANCA(A/T)AGG
XpsD_EcoRI_F	GAATTCCAAGGCCGAAAAAGTCTCTG
XpsD_EcoRI_R	GAATTCCACCAGCAGGGTATTGGTCT

Restriction sites incorporated into primers are underlined.

**Table 3:** Hydrolysis of CMC by wild-type and mutant strains.

*X. axonopodis* pv. *citri* strain	CMCase activity index^*, #^	Standard deviation
Wild type	5.07a^&^ ± 0.1487	0.7285
XpsD-defective mutant (30E9)	0.86b ± 0.0804	0.3941

^*^(H^2^- C^2^)/ C^2^ (H - diameter of the halo, C - diameter of the colony). ^#^Average ± standard error of the means (n = 24). ^&^Statistically significant differences (p < 0.001), as determined by the Student *t* test, are indicated by letters “a” and “b” for comparison to the strains.
